# Analysis of Mental Health Disease Trends Using BeGraph Software in Spanish Health Care Centers: Case Study

**DOI:** 10.2196/15527

**Published:** 2021-06-16

**Authors:** Susel Góngora Alonso, Andrés de Bustos Molina, Beatriz Sainz-De-Abajo, Manuel Franco-Martín, Isabel De la Torre Díez

**Affiliations:** 1 Department of Signal Theory and Communications, and Telematics Engineering University of Valladolid Valladolid Spain; 2 Next Limit Technologies Madrid Spain; 3 Department of Technology-CIEMAT Madrid Spain; 4 Psychiatry Department Rio Hortega University Hospital and Zamora Hospital Valladolid, Zamora Spain

**Keywords:** BeGraph software, diseases, health care complexes, mental health, visualization

## Abstract

**Background:**

In the era of big data, networks are becoming a popular factor in the field of data analysis. Networks are part of the main structure of BeGraph software, which is a 3D visualization application dedicated to the analysis of complex networks.

**Objective:**

The main objective of this research was to visually analyze tendencies of mental health diseases in a region of Spain, using the BeGraph software, in order to make the most appropriate health-related decisions in each case.

**Methods:**

For the study, a database was used with 13,531 records of patients with mental health disorders in three acute medical units from different health care complexes in a region of Spain. For the analysis, BeGraph software was applied. It is a web-based 3D visualization tool that allows the exploration and analysis of data through complex networks.

**Results:**

The results obtained with the BeGraph software allowed us to determine the main disease in each of the health care complexes evaluated. We noted 6.50% (463/7118) of admissions involving unspecified paranoid schizophrenia at the University Clinic of Valladolid, 9.62% (397/4128) of admissions involving chronic paranoid schizophrenia with acute exacerbation at the Zamora Hospital, and 8.84% (202/2285) of admissions involving dysthymic disorder at the Rio Hortega Hospital in Valladolid.

**Conclusions:**

The data analysis allowed us to focus on the main diseases detected in the health care complexes evaluated in order to analyze the behavior of disorders and help in diagnosis and treatment.

## Introduction

Currently, the medical care provided in a wide range of clinical applications makes use of the latest technologies, multimedia data, multidimensional medical images, videos, and data based on sensors and texts [1]. In the area of mental health, algorithms and data mining techniques are used to extract large volumes of predictive data, build models based on large-scale medical information, and predict diagnoses [2]. Despite its advantages in the medical field, these techniques and algorithms pose a computational challenge. Complex data management and software design are necessary [3,4]. Medical applications linked to the network sciences, which are responsible for collecting, managing, analyzing, interpreting, and presenting data, allow the development of visualization tools that support diagnosis and medical decision making [5,6].

Today, we have web-based 3D visualization tools that allow the exploration and analysis of data [7,8]. BeGraph is a computing application, in the cloud, dedicated to the visualization and analysis of complex networks for companies and the scientific field. Networks are becoming popular in the field of data analysis and big data because they contain information that other data structures lack [9]. Characteristics, such as the phenomenon of the small world, the diffusion of events, and the grouping in clusters based on communities, are exclusive of networks. Additionally, networks are the basic data structure in BeGraph. A network is a set of N entities called nodes related by a set of L links that represent binary relationships. Nodes and links can have associated properties, either imported from a data source or calculated with BeGraph [10].

The BeGraph software uses different metrics that consist of mathematical operations based on the graphics theory used to characterize the network. These metrics provide information about the centrality of the nodes, busiest links, cohesion properties, grouping of networks, and topology [11,12].

The purpose of this research was to analyze the trend of mental health illnesses in a region of Spain through BeGraph in order to make health-related decisions, thus helping medical personnel to focus their research on preventing and improving the life quality of patients most affected by the most common diseases.

There are similar studies that show the viability of our research. In a previous study [1], the authors presented a 3D medical graphical avatar (MGA). It is a web-based personal health record visualization system designed to explore web-based delivery of a wide array of medical data types including multidimensional medical images, medical videos, text-based data, and spatial annotations. In a previous report [7], the authors developed an interactive 3D tool to facilitate the visualization and exploration of covariate distributions and imbalances across evidence for network meta-analysis (NMA). In another report [13], researchers developed 3D molecular dynamics visualization (MDV) that offers an immersive viewing experience of biomolecular systems.

Below, we provide a description of BeGraph, the methodology used, the results obtained in the study, and the discussion and conclusions of the investigation.

## Methods

### Database

For the study, the data set used had a total of 13,531 records of patients with mental health disorders. The data were provided by the following three acute care units of the care centers: University Clinic of Valladolid (7118 records), Zamora Hospital (4128 records), and Rio Hortega Hospital (2285 records). These health care complexes are located in the region of Castilla y Leon, Spain. The records of the acute units were used because they contain the largest amount of data. Moreover, patients with mental disorders are admitted to acute units at moments of greatest severity of their pathologies; therefore, suitable management of the acute phase is essential for disease stabilization. All the data included in the study were anonymous and followed the Ninth Revision, International Classification of Diseases (ICD-9), which the World Health Organization established to track mortality statistics, and they corresponded to the period between 2005 and 2015. The database collects information such as dates of hospital admission and departure, days of stay, year of registration, health care complex, gender, psychiatric diseases, other diseases not belonging to the area of mental health, and therapies corresponding to each medical diagnosis. Among all the data collected in the database for the study, the variables included were (1) the diagnoses of the mental health area, (2) the gender of the admitted patients, (3) the year of admission, and (4) the health care complex to which the patients belong.

The irrelevant variables for the study were eliminated, and these were (1) therapies, (2) other medical diagnoses that do not belong to the mental health area, (3) dates of admission, and (4) discharge from the hospital. Null values, double blanks, and special characters were also eliminated to avoid false categories.

### BeGraph Software

BeGraph is a cloud platform for the visualization and analysis of complex networks. It is efficient and highly scalable, being able to represent, in 3D, networks of several million links. For this, it uses the layout algorithms implemented. It allows exploration and interaction with the network in real time, allowing the analyst to become familiar with the data and obtain qualitative information quickly and easily. The 3D visualization of the network is highly configurable in order to simultaneously represent various characteristics of the nodes or links (depending on shape, color, or size). In this way, researchers can visually identify the clusters or communities and patterns of the network [10].

BeGraph allows the loading of properties of nodes and links, which can influence the results of the metrics typical of graph theory. There are about 20 different metrics that characterize the network following criteria such as topology, centrality, cohesion, and clustering or division into communities. These metrics are characteristics of graph theory and provide information that conventional statistics do not provide. In the following section, we describe the database, metrics, and algorithms used in this study.

#### BeGraph Algorithms

As the BeGraph software is a cloud application, interaction with the user is done through a web browser. Input data are uploaded to the cloud (.csv files or remote databases), and the metrics are configured and executed. Several complex calculations can be made in parallel.

All these calculations take place on cloud servers transparent to the user. Additionally, the cloud server hardware can be configured to adapt to the size of the network for analysis. On the user’s local computer, only the calculations related to the rendering processes of visualization take place. Depending on the number of nodes and links, these computations can be very expensive. It is necessary to have a powerful graphics processing unit (GPU) to visualize and navigate through large networks.

Once the network is loaded on the platform, it is necessary to use a layout algorithm to represent and visualize the network. The layout algorithms in networks have the nodes in the plane or space for their visualization. In the arrangement of each node (embedding), an attempt is made to preserve the distance between nodes in the network in the Euclidean space. Thus, the nodes that are neighbors or are a few jumps between them will be found nearby in the visualization. The layout algorithms preserve the connectivity and structure of network clusters, making them obvious to the observer. Among the available algorithms, we used the so-called Force Atlas that considers the network as a system of N bodies (nodes) on which the below forces act [14].

*Repulsive force:* It is assumed that all nodes are spheres with a charge of the same sign that are repelled according to Coulomb law. Therefore, for any pair of nodes (*u* and *v*) we will have the following:

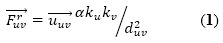



where *k_u_* and *k_v_* are the degrees of the nodes and α is a constant adjusted by the user.

*Attraction force:* We consider that the nodes connected by a link are attracted with a force proportional to the distance that separates them as if they were joined by a spring (Hooke law) as follows:

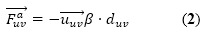



where β is manually adjusted.

*Gravity force:* To avoid the not connected components of the network being separated too much, it is possible to adjust a small central force (according to the parameter λ) that attracts the nodes to the origin of coordinates as follows:

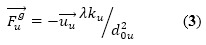



where *d*_0u_^2^ is the distance from the node to the origin of coordinates.

The combination of these three forces (with various acceleration algorithms, such as the Barnes-Hut approach to avoid calculating O [N2] repulsive forces) spatially provides the nodes in an intelligent and illustrative manner, revealing topological characteristics of the network.

Various metrics (graph theory) were used in this study. The first is *degree centrality*. It is the most basic measure of centrality of the network and is simply the number of neighbors of a node. It represents locally the importance of a node in the network, taking as a criterion the number of connections it has. The second is *eigenvector centrality*. It does a ranking of nodes in a nonlocal way, taking into account the degree of its neighbors, the neighbors of its neighbors, and so on. It reveals nodes that, without having a high degree, are important in the network because they are connected or close to other high-grade nodes. The third is *louvain communities*. Communities in a graph or network are the equivalent of clusters in other data structures. They are sets of nodes connected more densely to each other than to the rest of the network. As the problem of partitioning a graph is NP completeness (nondeterministic polynomial-time complete), community detection algorithms are limited by the size of the network, or they produce approximate results. We used the well-known algorithm of Louvain [12] for its efficiency and the quality of its results.

## Results

In this section, we present the results using a database that included a total of 13,531 anonymous medical records. We used BeGraph software, which allows the user to upload, store, and share several networks in the cloud. Each network can be viewed and explored using a web browser, giving a general idea of its structure and topological properties [10].

To obtain the results shown below, we used different metrics and one of the BeGraph design algorithms (Force Atlas Layout) [14]. It is an algorithm based on a system directed by force and optimized to deal with large networks, and it is highly configurable. *Degree centrality* is responsible for identifying the most popular nodes in the network. The size of the node and the warmth of the color represent the degree of each node. *Eigenvector centrality* is responsible for measuring the centrality of the selected node, normalizing the components of the eigenvector to a maximum value of 1.

With the 3D visualization tool, we explored the data and represented the results. We seek to clarify aspects such as (1) networks of diseases prevalent in the 2005-2015 period of patient admission records and (2) networks of prevalent diseases depending on the patient’s gender, with consideration of each of the health care complexes included in the study.

**Figure 1:**
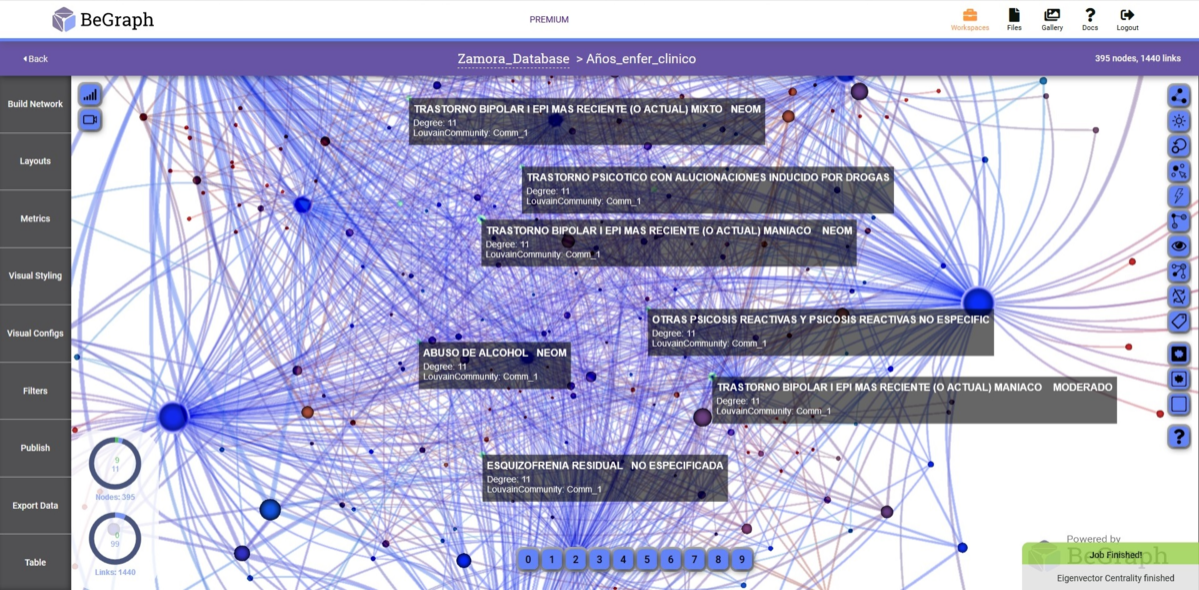
Network with prevalent diseases-years in the University Clinic of Valladolid.

In Figure 1, the prevalent diseases of the University Clinic of Valladolid are shown during the period from 2005 to 2015.

**Figure 2:**
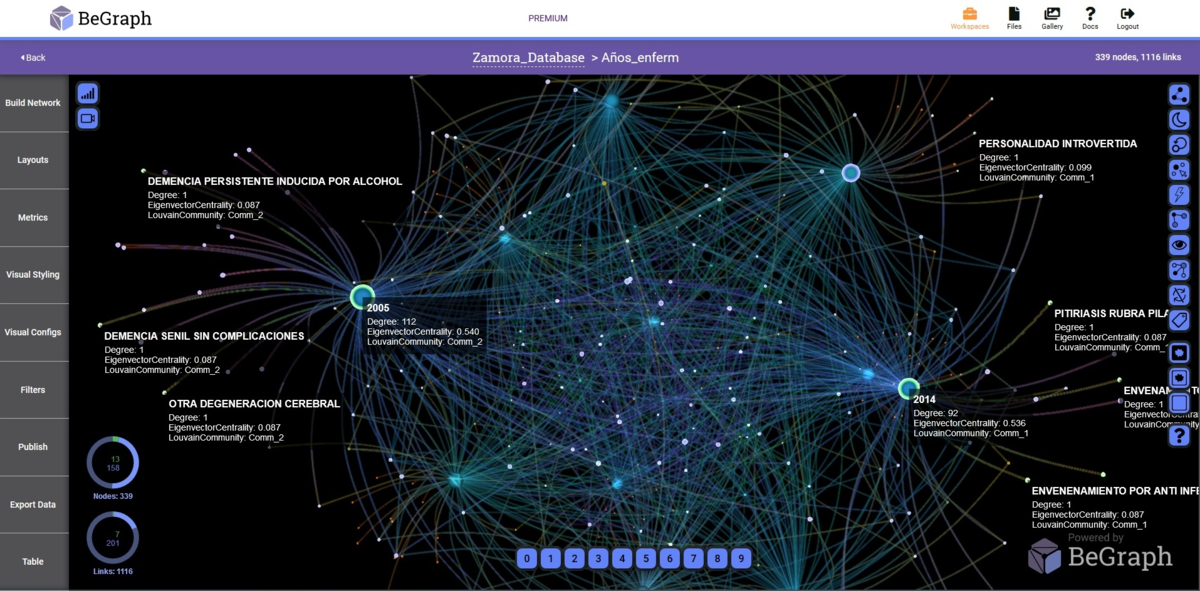
Network with prevalent diseases-years in the Assistance Complex of Zamora.

**Figure 3:**
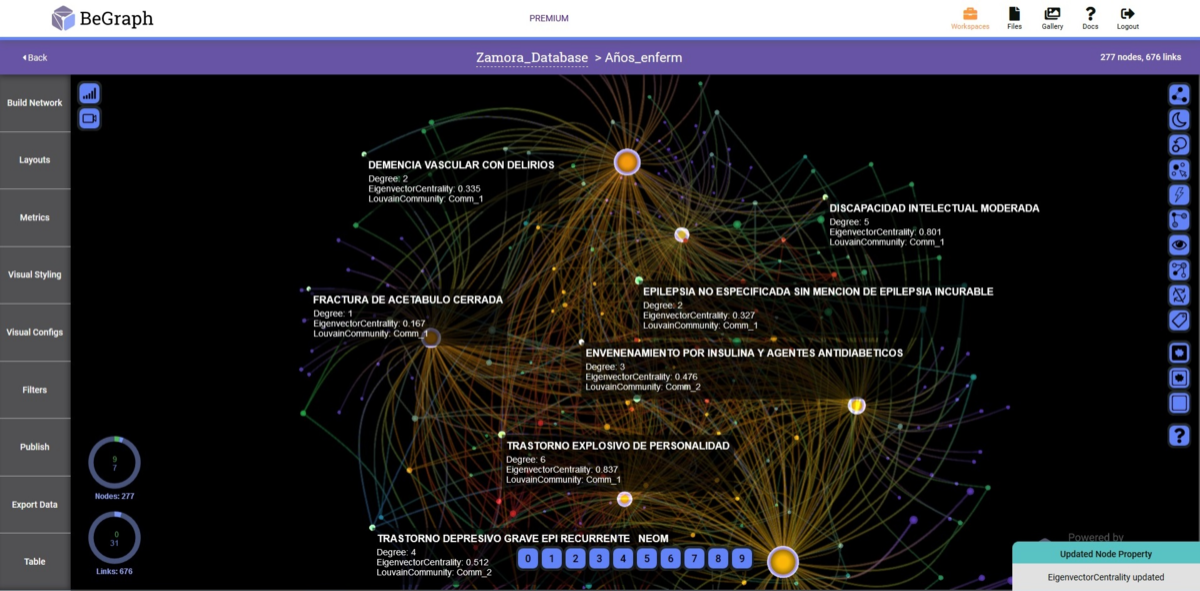
Network with prevalent diseases-years in the Rio Hortega Hospital of Valladolid.

The nodes are identified with a property called NodeLabel, the ubiquitous node property used to identify the nodes in metrics and filters. The largest nodes represent the year and the smallest the diseases detected in that hospital. The links represent the binary relationships between them. In Figures 1, 2, and 3, it can be seen that there are diseases that were only registered in a given year and not present in the rest of the years. The nodes of the diseases that are interrelated with more than 1 year represent the same type of disease registered in several years.

In Figures 2 and 3, the networks with prevalent diseases in the Health Care Complex of Zamora and the Rio Hortega Hospital, respectively, are shown. The period analyzed is from 2005 to 2015 and visually reflects the relationship of the data, providing medical personnel with criteria for decision making.

The results of the main diseases presented by patient admission records with mental health diseases in the different health care complexes are shown in Table 1.

**Table 1:** Admission records according to mental health diseases for health care complexes in Valladolid and Zamora.

Main mental health diseases	Admission records, n (%)			
	University Clinic of Valladolid (N=7118)	Zamora Hospital (N=4128)	Rio Hortega Hospital of Valladolid (N=2285)	
Unspecified paranoid schizophrenia	463 (6.50%)	10 (0.24%)	15 (0.66%)	
Adaptation disorder with mixed disturbance of emotions and behavior	457 (6.42%)	7 (0.17%)	18 (0.79%)	
Another alcohol dependence and neom	372 (5.22%)	N/A^a^	N/A	
Mixed adaptation disorder of anxiety and depressed humor	356 (5.00%)	95 (2.30%)	36 (1.58%)	
Unspecified psychosis	352 (4.94%)	45 (1.09%)	87 (3.81%)	
Dysthymic disorder	289 (4.06%)	161 (3.90%)	202 (8.84%)	
Chronic paranoid schizophrenia with acute exacerbation	3 (0.04%)	397 (9.62%)	108 (4.73%)	
Chronic schizoaffective disorder/acute exacerbation	N/A	209 (5.06%)	74 (3.24%)	
Another alcohol dependence and continuous neom	13 (0.18%)	173 (4.19%)	183 (8.01%)	
Poisoning by tranquilizers based on benzodiazepine	113 (1.59%)	81 (1.96%)	110 (4.81%)	
Delirious disorder	271 (3.81%)	114 (2.76%)	91 (3.98%)	

^a^N/A: not applicable.

The percentages are obtained from the total records. The University Clinic of Valladolid had 7118 records, Zamora Hospital had 4128 records, and Rio Hortega Hospital had 2285 records.

**Figure 4:**
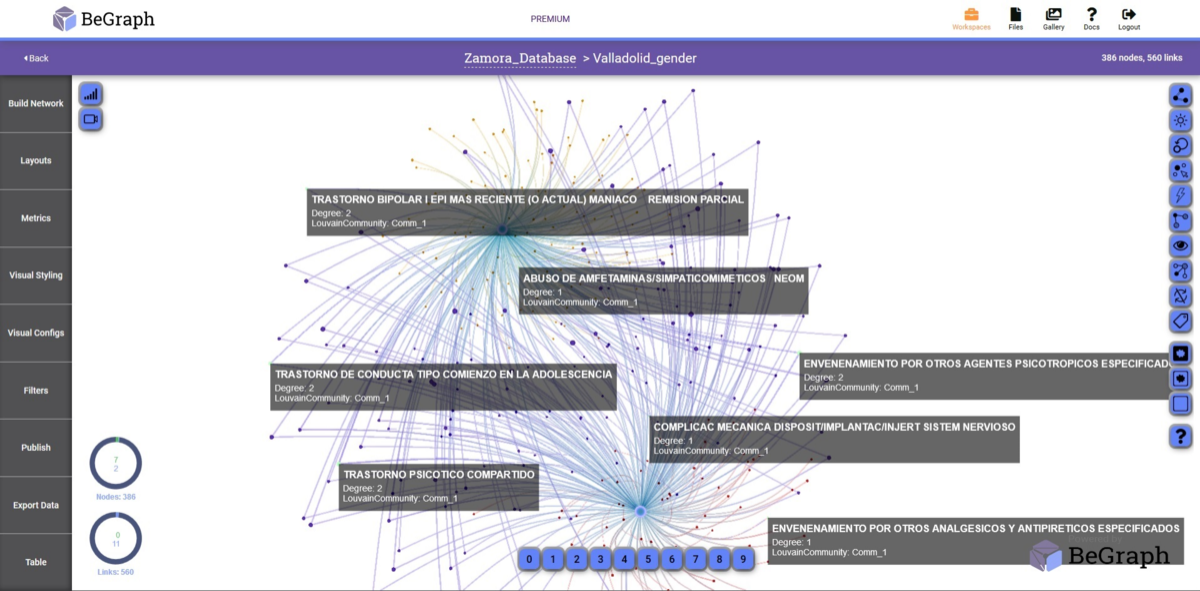
Network with prevalent diseases-gender in the University Clinic of Valladolid.

**Figure 5:**
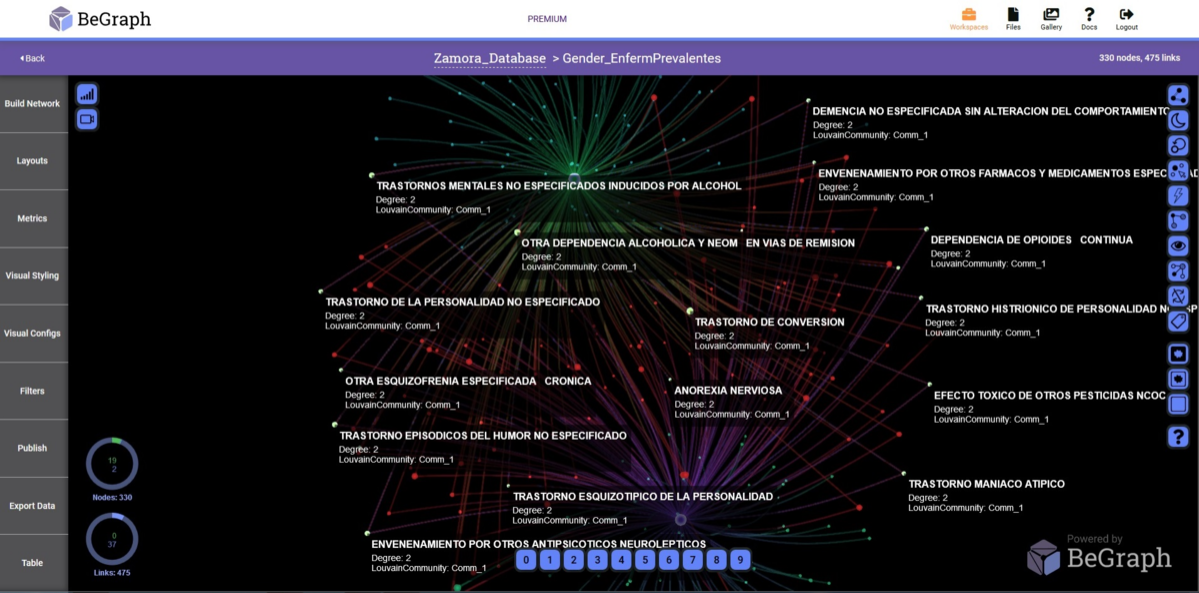
Network with prevalent diseases-gender in the Healthcare Complex of Zamora.

**Figure 6:**
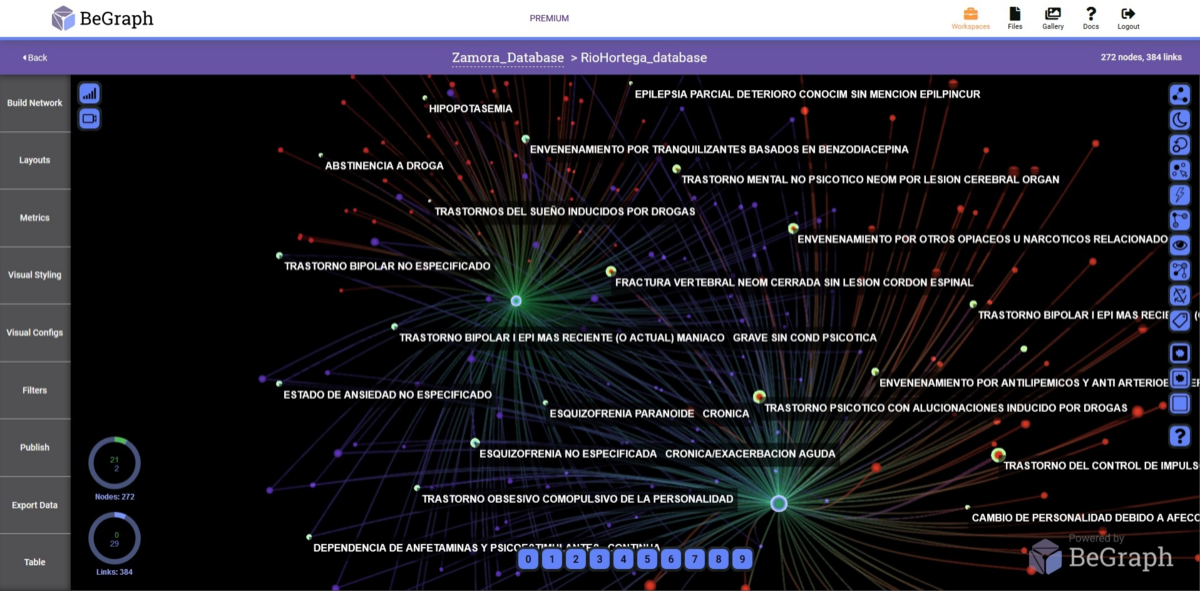
Network with prevalent diseases-gender in the Rio Hortega Hospital of Valladolid.

Another parameter analyzed in our study was prevalent diseases depending on the patient’s gender, as shown in Figures 4, 5, and 6.

In Figures 4 and 5, the main nodes represent the gender of the admission records. The upper node corresponds to men and the lower node corresponds to women. The small nodes represent the diseases detected in the University Clinic of Valladolid and Zamora Hospital, respectively. In the Rio Hortega Hospital, the upper node corresponds to women and the lower node corresponds to men (Figure 6). The diseases are visually shown only in men and women, and the common ones among them are displayed.

Tables 2 and 3 present a summary of the main diseases detected in each of the health care complexes, with consideration of gender.

**Table 2:** Admission records according to mental health diseases in males for health care complexes in Valladolid and Zamora.

Main mental health diseases in males	Admission records, n (%)			
	University Clinic of Valladolid (N=3558)	Zamora Hospital (N=2282)	Rio Hortega Hospital of Valladolid (N=1150)	
Unspecified paranoid schizophrenia	337 (9.47%)	6 (0.26%)	14 (1.22%)	
Another alcohol dependence and neom	273 (7.67%)	N/A^a^	1 (0.09%)	
Adaptation disorder with mixed disturbance of emotions and behavior	200 (5.62%)	N/A	10 (0.87%)	
Chronic paranoid schizophrenia with acute exacerbation	3 (0.08%)	307 (13.45%)	74 (6.43%)	
Another alcohol dependence and continuous neom	7 (0.20%)	139 (6.09%)	136 (11.83%)	
Chronic schizoaffective disorder/acute exacerbation	N/A	82 (3.59%)	34 (2.96%)	
Dysthymic disorder	64 (1.80%)	31 (1.36%)	55 (4.78%)	

^a^N/A: not applicable.

**Table 3:** Admission records according to mental health diseases in females for health care complexes in Valladolid and Zamora.

Main mental health diseases in females	Admission records, n (%)		
	University Clinic of Valladolid (N=3560)	Zamora Hospital (N=1846)	Rio Hortega Hospital of Valladolid (N=1135)
Adaptation disorder with mixed disturbance of emotions and behavior	257 (7.22%)	7 (0.38%)	8 (0.70%)
Dysthymic disorder	225 (6.32%)	130 (7.04%)	147 (12.95%)
Mixed adaptation disorder of anxiety and depressed humor	202 (5.67%)	48 (2.60%)	20 (1.76%)
Chronic schizoaffective disorder/acute exacerbation	N/A^a^	127 (6.88%)	40 (3.52%)
Chronic paranoid schizophrenia with acute exacerbation	N/A	90 (4.88%)	34 (3.00%)
Poisoning by tranquilizers based on benzodiazepine	73 (2.05%)	57 (3.09%)	64 (5.64%)
Disorder or unspecified dissociative reaction	35 (0.98%)	16 (0.87%)	49 (4.32%)

^a^N/A: not applicable.

The results presented in Tables 2 and Table 3 show considerable differences in the types of disorders. Men have disorders related to schizophrenia and alcohol, while women have dysthymic and adaptive disorders.

To obtain the different networks shown in Figures 1-6, we used the layout algorithm called Force Atlas, which considers the network as a system of N nodes on which different forces act. In the algorithm configuration, we used 500 iterations, gravity of 1, jitter tolerance of 0.5, and mass range of 10, while in the configuration of the metrics, the *eigenvector centrality* used 100 iterations. These parameters allowed us to obtain the best visualizations of the networks formed by the data.

## Discussion

In this study, we performed a visual analysis of mental health diseases prevalent in a region of Spain, using the BeGraph software, in order to make health-related decisions. The results show that BeGraph is a tool that allows visualization, in 3D, of the behavior of a network, offering complementary information to classical statistical analysis. As a novelty, we presented the analysis and visualization of medical data from the point of view of the theory of complex networks. Metrics based on networks or graphs provide new properties, which can be used to describe the data quantitatively or as additional properties in other machine learning algorithms. The visualization of the network provides an interactive method of scanning medical data as well as qualitative characterization quickly and easily.

In this work, we used a database of 13,531 medical records. The analysis allowed us to determine the main disease detected in each of the health care complexes. We noted 6.50% (463/7118) of admissions involving unspecified paranoid schizophrenia at the University Clinic of Valladolid, 9.62% (397/4128) of admissions involving chronic paranoid schizophrenia with acute exacerbation at the Zamora Hospital, and 8.84% (202/2285) of admissions involving dysthymic disorder at the Rio Hortega Hospital. In the health care complexes with the most admissions, such as the University Clinic of Valladolid and Zamora Hospital, the greatest mental health disorder was presented by patients with different types of schizophrenia. These data allowed us to focus on this disease to analyze its behavior and help in diagnosis and treatment. In Rio Hortega Hospital, dysthymic disorder represented the main disease. It was present in other health care complexes with similar amounts of admissions, but represented a lower value with regard to the main diseases detected in the University Clinic of Valladolid.

The analysis of the main diseases depended on gender. The results of the disorders were different in men and women. In the case of men, in the three health care complexes, the highest number of admissions detected was related to schizophrenia disorders and alcohol dependence. In women, the disorders were based on dysthymic disorders, adaptive disorders, schizoaffective disorders, and poisoning. Therefore, the results indicated that it is necessary to take gender into account in order to make decisions in diagnoses. The trends shown by these results provide a basis for future research regarding the prediction of mental health diseases and common patterns among patients with the same diseases. In this case, we focused on schizophrenia disorders taking into account gender, stay days, health care complex, age, and other diagnosed diseases. We propose the prediction of patient readmissions with schizophrenia using machine learning techniques.
